# Magnolol-mediated regulation of plasma triglyceride through affecting lipoprotein lipase activity in apolipoprotein A5 knock-in mice

**DOI:** 10.1371/journal.pone.0192740

**Published:** 2018-02-09

**Authors:** Chun-Kai Chang, Xiu-Ru Lin, Yen-Lin Lin, Woei-Horng Fang, Shu-Wha Lin, Sui-Yuan Chang, Jau-Tsuen Kao

**Affiliations:** 1 Department of Clinical Laboratory Sciences and Medical Biotechnology, College of Medicine, National Taiwan University, Taipei, Taiwan; 2 Department of Laboratory Medicine, National Taiwan University Hospital, Taipei, Taiwan; 3 Department of Laboratory Medicine, National Yang-Ming University Hospital, Yilan, Taiwan; Beijing Key Laboratory of Diabetes Prevention and Research, CHINA

## Abstract

Hyperlipidemia is a risk factor of arteriosclerosis, stroke, and other coronary heart disease, which has been shown to correlate with single nucleotide polymorphisms of genes essential for lipid metabolism, such as *lipoprotein lipase (LPL)* and *apolipoprotein A5 (APOA5)*. In this study, the effect of magnolol, the main active component extracted from *Magnolia officinalis*, on LPL activity was investigated. A dose-dependent up-regulation of LPL activity, possibly through increasing *LPL* mRNA transcription, was observed in mouse 3T3-L1 pre-adipocytes cultured in the presence of magnolol for 6 days. Subsequently, a transgenic knock-in mice carrying APOA5 c.553G>T variant was established and then fed with corn oil with or without magnolol for four days. The baseline plasma triglyceride levels in transgenic knock-in mice were higher than those in wild-type mice, with the highest increase occurred in homozygous transgenic mice (106 mg/dL vs 51 mg/dL, p<0.01). After the induction of hyperglyceridemia along with the administration of magnolol, the plasma triglyceride level in heterozygous transgenic mice was significantly reduced by half. In summary, magnolol could effectively lower the plasma triglyceride levels in APOA5 c.553G>T variant carrier mice and facilitate the triglyceride metabolism in postprandial hypertriglyceridemia.

## Introduction

The role of hypertriglyceridemia as a risk factor of coronary heart disease remains controversial. However, emerging evidence points to an association between elevated serum triglycerides and coronary heart disease [1–4]. Elevated blood triglyceride is a common metabolic disorder in the general population. Although it can be caused by many factors, a myriad of individuals have a genetic tendency, and numerous genes responsible for variation in triglyceride levels have also been explicated. One of the genes is *apolipoprotein A5*. Transgenic knock-in mice overexpressing human apolipoprotein A5 decreased plasma triglyceride levels to one-third of those in control mice; conversely, knock-out mice lacking *APOA5* had four times as much plasma triglycerides as controls [5]. In addition to the whole *APOA5* gene, a couple of important single nucleotide polymorphisms (SNPs) and haplotypes with a widely confirmed effect on plasma triglycerides concentrations have been described [6–8]. Our previous report described an *APOA5* variant, c.553G>T (rs2075291, Gly185 > Cys) that is associated with hypertriglyceridemia [9]. Individuals carrying the 553T allele were found to have odds of 11.73 of developing hypertriglyceridemia in comparison with individuals with the major allele. Moreover, the minor T allele at this residue was significantly associated with increased risk of coronary artery disease after adjustment for common cardiovascular risk factors [10]. APOA5 residue 185Gly was very important in LPL-mediated very-low-density lipoproteins (VLDL) hydrolysis [11].

Magnolol is one of the active components from *Magnolia officinalis*, a widely used traditional Chinese medicine [12]. Magnolol stimulated lipolysis of lipid-laden macrophages [13]. Magnolol was also reported to *in vitro* enhance adipocyte differentiation and glucose uptake in 3T3-L1 cells, and improve insulin sensitivity through the activation of PPARγ as a ligand [14, 15]. Moreover, magnolol also induced the expression of LPL and adiponectin in cell culture system [14].

LPL, which hydrolyzes triglycerides (TG) in lipoprotein and promotes cellular uptake of chylomicron remnants and free fatty acid, is important in lipid metabolism [16]. Magnolol can regulate the expression of lipoprotein lipase *in vitro*; however, whether it has an effect on lipid metabolism *in vivo*, in particular of plasma triglycerides levels, is unknown.

In this study, we used pre-adipocyte 3T3-L1 cells and established a transgenic knock-in mice carrying c.553G>T variation on *APOA5* gene to evaluate the effect of magnolol on lipid metabolism *in vitro* and *in vivo*. Our data indicate that magnolol could increase LPL activity in cells and reduce the plasma triglyceride level in *APOA5* c.553G>T variant carrier mice and facilitate triglyceride metabolism in mild hypertriglyceridemia. These findings suggest the possible use of magnolol to lower the lipid levels in mild hypertriglyceridemia in patients carrying c.553G>T variation on *APOA5* gene.

## Materials and methods

### Cell culture

Mouse 3T3-L1 pre-adipocytes purchased from the Bioresource Collection and Research Center (BCRC #60159; Hsinchu, Taiwan) were grown in Dubecco's modified Eagle's medium (DMEM) (Sigma, Amherst, NJ, USA) supplemented with 10% bovine calf serum (Hyclone, Utah, USA), antibiotics (100 U/ml penicillin and 100 μg/ml streptomycin (Gibco BRL, NY, USA) at 37 °C under a humidified 5% CO_2_ atmosphere. To investigate the effect of magnolol on LPL expression and whether this effect is via PPAR-mediated activation of LPL gene expression, different concentrations of magnolol (Sigma, Amherst, NJ, USA), GW9662 (Sigma, Amherst, NJ, USA) and MK886 (Sigma, Amherst, NJ, USA), were added into culture medium and the cells were cultured for 6 days. One hour before harvest, 30 U/mL of heparin (Sigma, Amherst, NJ, USA) were added. Then the cells were washed with phosphate buffered saline (PBS) solution (One-Star Biotechnology Co., Ltd., Taipei, Taiwan) after the culture medium was collected. The cells pellets were resuspended in the reporter lysis buffer (Promega, Madison, WI, USA) and lysed by sonication.

### LPL activity assay

LPL activity of harvested culture medium and cell lysate was measured using the method reported by Fruchart-Najib [17]. Briefly, microtiter plate wells were loaded with 6 μL of normal pooled serum and 21.5 μg of VLDL-TG from normal pooled serum. After making up the final volume to 15 μL with PBS, then either 15 μL of culture medium or cell lysates were added. After 1 hour incubation at 37°C, 50 μL of stop solution (50 mM KH2PO4 and 0.1% Triton X-100, pH 6.9) was added. The free fatty acid liberated was measured using Clinimate NEFA kit (Daiichi, Japan).

### RNA preparation and quantitative real-time PCR

Total RNA was isolated from mouse 3T3-L1 pre-adipocytes using TRI REAGENT^™^ (Sigma, Amherst, NJ, USA) and 5 μg total RNA from each sample was reverse-transcribed to cDNA according to the protocol of the reverse transcript system (Promega, USA) using oligo (dT)_18_ primers. After cDNA synthesis, quantitative real-time PCR was performed using the ABI Prism 7500 instrument (Applied Biosystems, Foster City, CA, USA) according to the manufacturer's protocol. The PCR condition were 1 cycle of 95°C for 3 min, followed by 40 cycles of 95°C for 15 s and 60°C for 30 s. The primer sequences used in PCR were as follows; LPL, 5’-CTG CTG GCG TAG CAG GAA GT-3’ and 5’-GCT GGA AAG TGC CTC CAT TG-3’; GAPDH, 5’-GCC AAA AGG GTC ATC ATC TC-3’ and 5’-GGC CAT CCA CAG TCT TCT-3’. Quantification was performed in duplicate and the experiments were repeated independently three times.

### Animal protocol

Animal protocols were approved by the Institutional Animal Care and Use Committee, NTUCM and NTUCPH of National Taiwan University in accordance with National Institutes of Health guidelines (IACUC application no. 20120286). All animals were housed at an ambient temperature with a 12 h light/dark cycle and were fed a standard rodent diet ad libitum and provided free access to water. The investigation and animal experiments conformed to the NIH guidelines, Guide for the care and use of laboratory animals (Publication No. 85–23, revised 2011 published by National Research Council).

### Construction of knock-in targeting vector and generation of human APOA5 knock-in mice

Recombineering-based method was used for generating knock-in mice [18]. Targeting DNA from BACs was subcloned into a high-copy plasmid vector, pL253. BACs contained 129S7/SvEv Brd-Hprt b-m2 strain mice *apoa5* gene were purchased from Sanger and were first transferred from its strain of origin (DH10B) into EL350 *E*. *coli*. The targeting vector was subsequently linearized and electroporated into 129/sv mice CJ7 ES cells. Southern blot analysis was used to screen the targeted clones of ES cells. The knock-in clones were further treated with the expression of *Flpe* to excise the *Neo* cassette and identified by Southern blotting. The final selected clones of ES cells were injected into mouse blastocysts (E3.5) to create the chimeras of human *APOA5* c.553G>T mutant knock-in mice. This experiment was performed by Gene Knockout Mouse Core, NTU Center of Genomic Medicine. The chimeric males were bred with C57BL/6 females to produce heterozygous mice. Mice were backcrossed 10 generations by crossing heterozygous male mice with heterozygous female mice to generate human *APOA5* c.553G>T homozygous mutant knock-in mice.

### Genotyping for the transgene

DNA prepared from tail clippings was analyzed by PCR using the primers described below. For amplification of human *APOA5*: mapoa5E1 forward: 5’-GAC CGA AAT AAG GAG CAA TCC AAC-3’; hAPOA5E2 reverse: 5’-GAG CCA TCT TCT GCT GAT GGA TC-3’. For amplification of mouse *apoa5*: mapoAV-F2 forward: 5’-ACA GTT GGA GCA AAG GCG TGA T-3’; mapoAV-R2 reverse: 5’-CTT GCT CGA AGC TGC CTT TCA G-3’. The PCR cycling conditions were 5 min denaturation at 95°C, followed by 40 cycles of 30 s at 95°C, 30 s at 60°C, and 30 s at 72°C. The PCR products were fractionated on 1.5% TAE agarose gel (UniRegion Bio-Tech, Taiwan) then stained with ethidium bromide for 20 min.

### Effect of magnolol on transgenic knock-in mice

To determine the effect of corn oil and magnolol on plasma triglyceride level in transgenic knock-in mice, a corn oil bolus (6 ml/kg) or magnolol (30 mg/kg; 30 μg/μL in corn oil) was administered to the male knock-in mice by oral gavage once daily for 4 days [19, 20]. On the fourth day, 2 h after feeding, knock-in mice were anesthetized using avertin (Fluka, Amherst, NJ, USA), then laparotomy was carried. Heparinized blood was collected via inferior vena cava for determination of biochemical analyses. Cervical dislocation was performed under anesthesia following the AVMA Guidelines for the euthanasia of animals (2013 edition).

### Biochemical analysis

The plasma total cholesterol, triglyceride, HDL cholesterol, aspartate aminotransferase (AST), alanine aminotransferase (ALT), total protein (TP), albumin (ALB), urea nitrogen (UN) and uric acid (UA) were measured on a Hitachi 7450 Analyzer (Hitachi, Japan) using Roche reagents.

### Statistical analysis

All data are representative of at least three independent experiments. Data are expressed as mean ± SD. Statistical analyses were performed using SPSS (ver. 12.0, Chicago, IL). Comparisons among groups were performed using ANOVA with post-hoc test (Scheffe method) or ANOVA with Mann-Whitney U test. A *p*<0.05 was considered statistically significant.

## Results

### Effect of magnolol on LPL activity in 3T3-L1 cells

**Fig 1:**
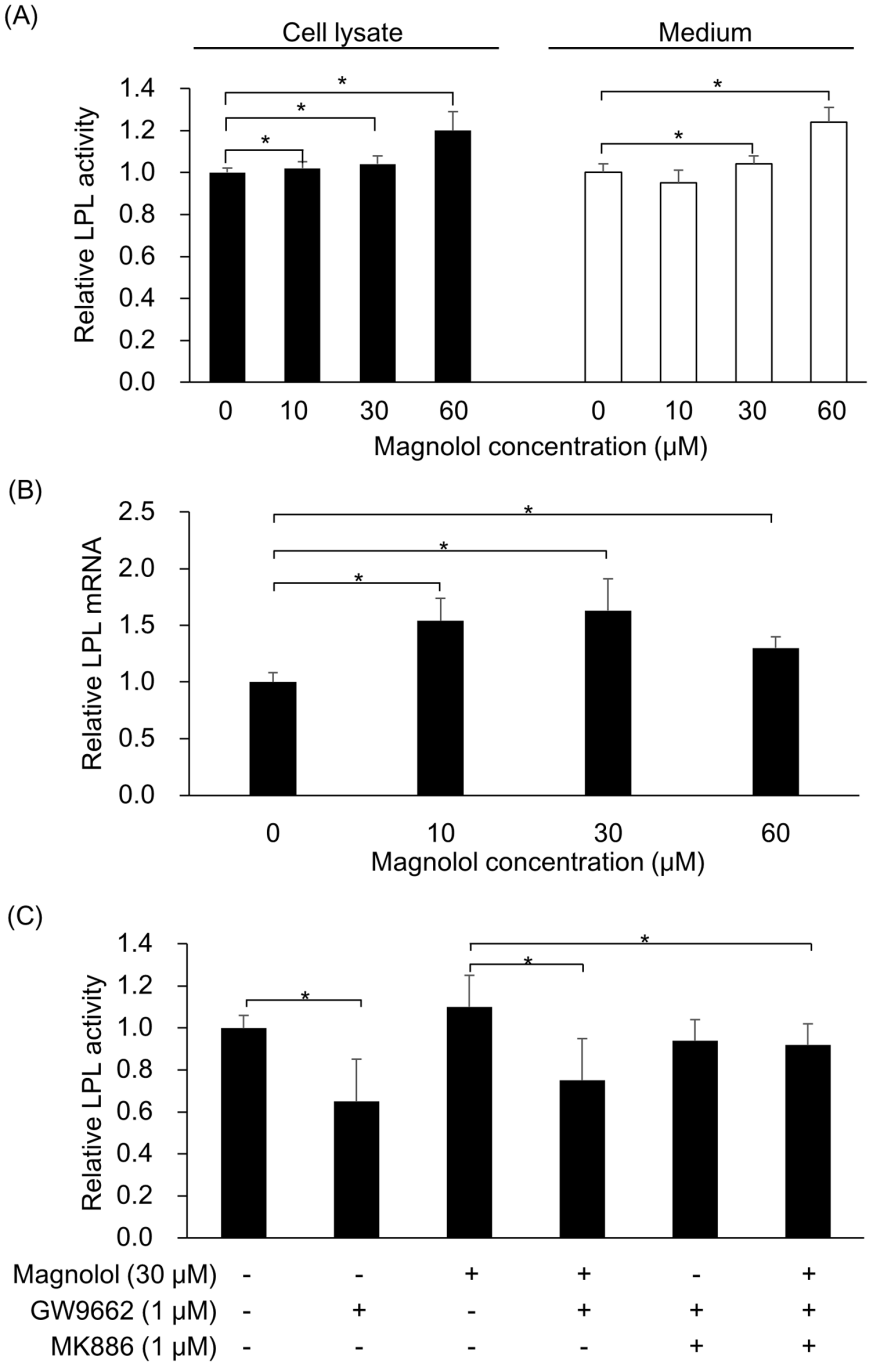
Effect of magnolol on LPL activity and gene expression in 3T3-L1 cells. (A) Magnolol dose-dependently increased the LPL activity. Mice 3T3-L1 pre-adipocytes were incubated in medium with/without the indicated concentration of magnolol for 6 days. LPL activity of harvested culture medium and cell lysate was measured using the method reported by Fruchart-Najib [17]. (B) Magnolol enhanced transcription of *LPL* gene. On day 6, total RNA was extracted and mRNA expression of adipocyte-specific genes was analyzed by real-time PCR. (C) The enhancement of LPL activity was suppressed by either PPARγ antagonist GW9662 or with PPARα antagonist MK886, respectively. All values are presented as means ± SD (n = 9) from three independent experiments. Statistical analysis was performed using ANOVA with post-hoc test (Scheffe method). *p<0.05.

In order to determine whether magnolol could affect LPL activity, mice pre-adipocyte 3T3-L1 cells were cultured with different concentrations of magnolol. Both cell lysates and cultured medium were harvested and examined for LPL activity after 6 days of incubation. As shown in Fig 1A, LPL activity in both cell lysates and supernatant was significantly increased about 1.04 ~ 1.25 fold along with the increased magnolol concentrations (*p*<0.05). This data showed that magnolol can up-regulate the activity of LPL with dose-dependence *in vitro*.

### The effect of magnolol on the gene expression of LPL

To determine the underlying mechanism of the induction of LPL activity by magnolol, the *LPL* mRNA levels in mice pre-adipocyte 3T3-L1 cells cultured with magnolol were measured (Fig 1B). The *LPL* mRNA expression was significantly enhanced about 1.3 ~ 1.63 fold in the presence of magnolol (*p*<0.05), and a weak dose-dependence was observed between the 0–30 μM magnolol concentrations (correlation coefficient, γ = 0.516, *p* = 0.008). To examine whether magnolol could activate PPARγ and subsequently lead to the elevated expression of *LPL* gene, magnolol and/or another two inhibitors, GW9662 and MK886 which inhibited PPARγ and PPARα respectively, were added to 3T3-L1 cells culture medium. The LPL activity was significantly decreased in the presence of GW9662. Meanwhile, the enhancement of LPL activity by magnolol was also suppressed in the presence of GW9662 alone or GW9662 and MK886 in combination (Fig 1C). These data implicated that the increase of LPL activity by magnolol could be attributed to PPARγ activation.

### Construction of knock-in targeting vector and generation of human APOA5 knock-in mice

To examine the influences of magnolol on lipid metabolism *in vivo*, a transgenic knock-in mice carrying *APOA5* c.553G>T (G185C) variant was established. The schematic design of the construction of knock-in targeting vectors and generation of human *APOA5* knock-in mice is shown in S1 Fig.

**Fig 2:**
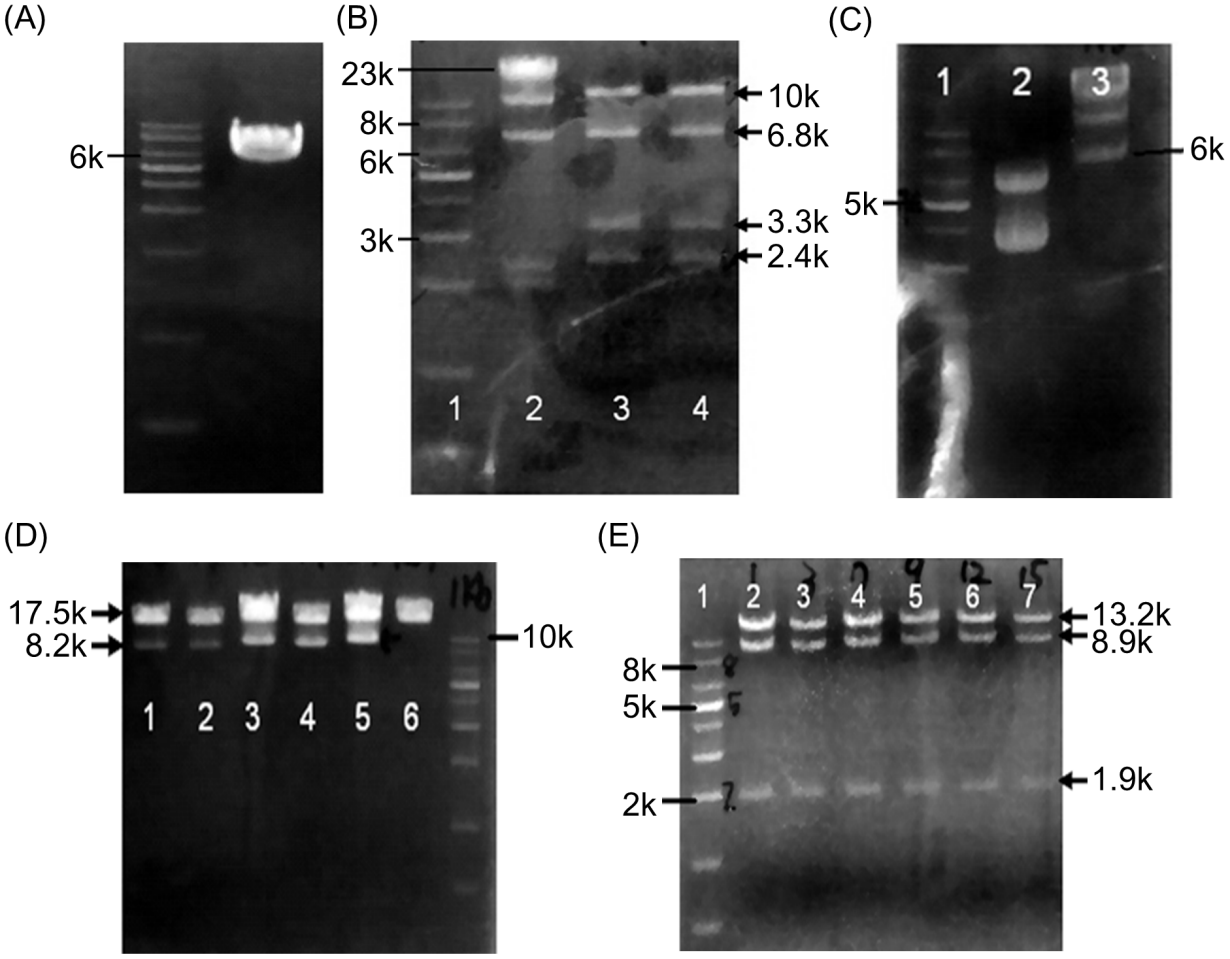
Electrophorogram of the targeting vectors. (A) Retrieved pL253 with homology arms, about 6.1 kb. (B) Retrieved A with about 17.7 kb. Lane 1: 1 kb marker; Lane 2: λ *Hind*III marker; Lane 3 and 4: retrieved A digested with *Kpn*I. (C) The pL452 with homology arms. Lane 1: 1 kb marker; Lane 2: pL452 with homology arms, about 5.4 kb (supercoid and linear form); Lane 3: λ *Hind*III marker. (D) First *lox*P site insertion Lane 1–5: retrieved A + vector C digested with *Spel*I; Lane 6: retrieved A + vector C; Last lane: 1kb marker. (E) Lane 1: 1kb marker; Lane 2–7: excision of *Neo* cassette and digested with *Xho*I.

Briefly, a 350 bp fragment PCR-amplified from BAC was subcloned into pL253 and used as the homology arms to produce the 6.1 kb plasmid (Fig 2A). The pL253 vector with homology arms was linearized and electroporated to BAC containing cells to produce 17.7 kb retrieved A (Fig 2B). A 300 bp fragment, containing *lox*P sites on both ends of neomycin resistance (*Neo*) cassette, was PCR-amplified from BAC and subcloned into pL452, which results in the 5.4 kb vector C plasmid (Fig 2C). Restricted enzyme digested vector C and retrieved A were co-transformed into EL350 *E*. *coli* to generate retrieved A + vector C (Fig 2D). After induction of Cre expression, the excision of the *Neo* cassette from the subcloned retrieved A + vector C was accomplished and the first *lox*P site was remained in the exon 4 of mice *apoa5* gene. The retrieved A + vector C—C was generated (Fig 2E).

**Fig 3:**
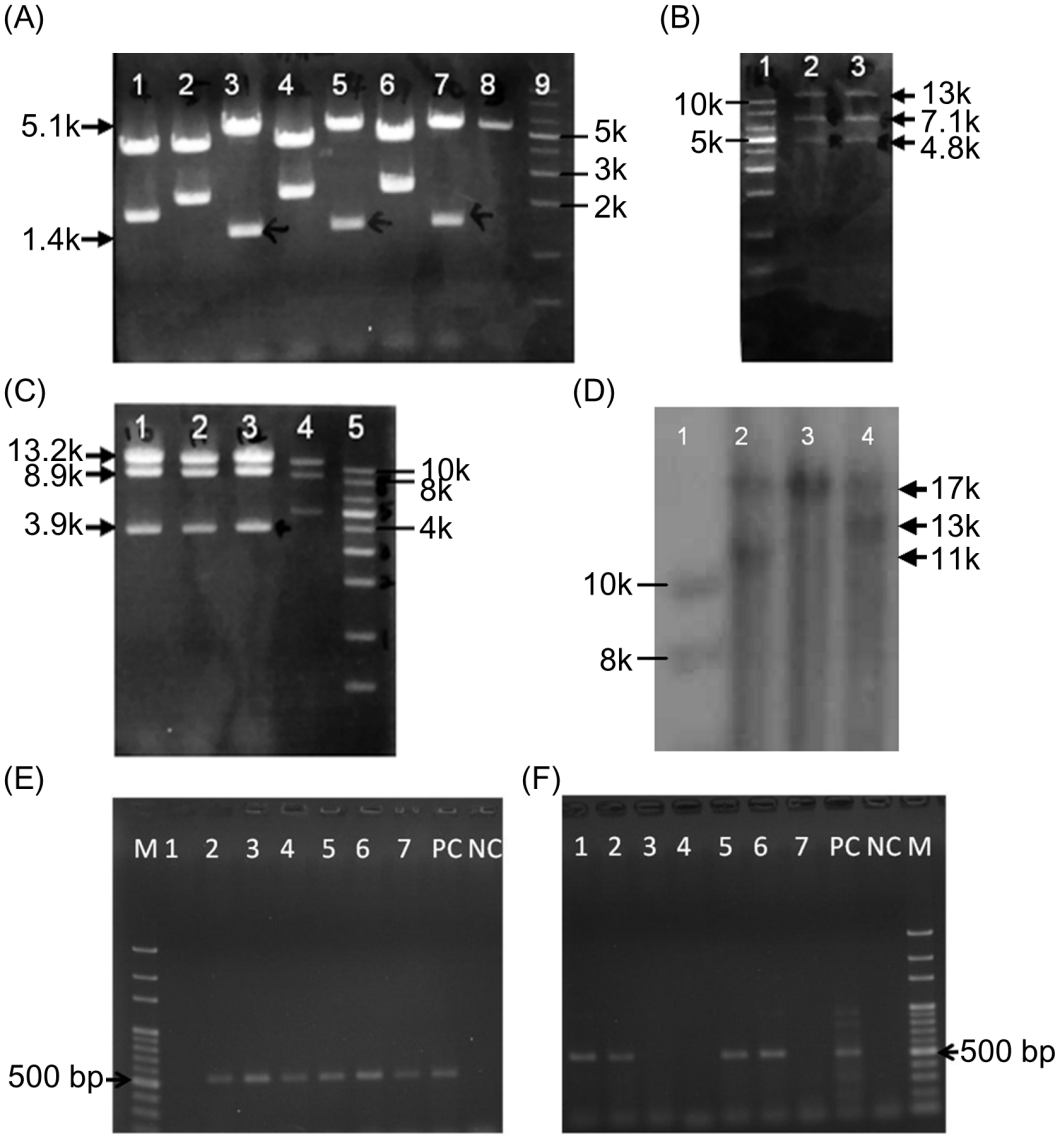
Constructs of targeting plasmids to knock-in human *APOA5* wild type and mutant genes and knockout mice endogenous *apoa5* gene. (A) Vector B. Lane 3, 5, 7: pL451 with human *APOA5* mini gene, digested with *Xmn*I; (B) Retrieved A + C—C + B, containing human *APOA5* mini gene 2 FRT sites and *Neo* cassette and mice *apoa5* gene, Lane 1: 1kb marker; Lane 2 and 3: retrieved A + C—C + B digested with *Apa*I; (C) Retrieved A + C—C + B—B. Lane 1–3: final constructs, excision of mice endogenous *apoa5* gene, digested with *Xho*I; Lane 4: retrieved A + C—C + B; Lane 5: 1kb marker. (D) Southern blotting of final construct. Lane 1: DNA marker (10kb, 8kb); Lane 2: excision of *Neo* cassette in human *APOA5* knock-in ES cell (17kb, 11kb); Lane 3: normal mouse ES cell (17kb); Lane 4: human *APOA5* knock-in ES cell (17kb, 13kb). (E) The genotype of human *APOA5* knock-in mice. Using the human *APOA5* primers, the size of product is 556 bp. Lanes 2, 3, 4, 5, 6 and 7 are positive for human *APOA5*. (F) Using the mouse *apoa5* primers, the size of product is 517 bp. Lanes 3, 4 and 7 do not contain mouse *apoa5* gene. M: 100 bp marker; PC: positive control; NC: negative control.

A 300 bp fragment, PCR-amplified from BAC, was subcloned into pL451. Human *APOA5* mini genes, which contained a fraction of intron, were first cloned into vector pCR3-Uni. After restriction enzyme digestion and ligation, the mini genes were subcloned into vector pL451 to generate vector B (Fig 3A). The human *APOA5* c.553G>T mutant vector B was constructed using the site-direct mutagenesis to introduce the point mutation. Restricted enzyme digested vector B and retrieved A + vector C—C were co-transformed into EL350 *E*. *coli* to generate the retrieved A + vector C—C + B plasmid (Fig 3B). After induction of Cre expression, the excision of the mice *apoa5* gene from the retrieved A + vector C—C + B was accomplished and the final constructs retrieved A + vector C—C + B—B was established (Fig 3C). The targeting vector was subsequently linearized and electroporated into 129/sv mice CJ7 ES cells. Southern blot analysis was used to screen the targeted clones of ES cells. The knock-in clones were further treated with the expression of *Flpe* to excise the *Neo* cassette and identified by Southern blotting (Fig 3D). The final selected clones of ES cells were injected into mouse blastocysts (E3.5) to create the chimeras of human *APOA5* c.553G>T mutant knock-in mice. The genotypes of knock-in mice are shown in Fig 3E and 3F.

### The association of APOA5 c.553G>T with elevated triglycerides on transgenic knock-in mice

After demonstrating magnolol could upregulate LPL activity *in vitro*, a transgenic knock-in mice carrying *APOA5* c.553G>T variant was established. Phenotypic analysis was first conducted to determine influence of *APOA5* c.553G>T variant on lipid metabolism. *APOA5* c.553G>T transgenic knock-in B6 mice were viable and fertile with no apparent gross abnormality. The transgenic knock-in male mice were divided into three groups including wild-type, heterozygous and homozygous, which carried no allele, one allele and two alleles of human *APOA5* c.553G>T variant, respectively. Mice were anesthetized and blood sample was collected with heparin. Subsequently, the plasma level of triglycerides (TG), cholesterol (CHO) and high-density lipoprotein (HDL-C) and other parameters were determined. As shown in Table 1, all three lipids in mice carrying *APOA5* c.553G>T variant were higher compared to those in wild-type mice (*p*<0.05), although no significant difference was observed in other parameters. In addition, both the plasma levels of triglycerides and high-density lipoprotein cholesterol exhibited a correlation with the number of alleles of *APOA5* c.553G>T variant carried in mice. This data confirmed that apolipoprotein A5 in transgenic knock-in mice with human *APOA5* c.553G>T variant could not efficiently activate LPL activity and led to the elevation of triglyceride concentrations.

**Table 1:** The association of *APOA5* c.553G>T with elevated triglycerides in 12-week-old transgenic knock-in mice.

	Wild-Type (n = 29)	Heterozygous (n = 31)	Homozygous (n = 16)
TG (mg/dL)	50.97±17.62	58.84±27.72^a^	105.99±61.63^a^
CHO (mg/dL)	59.31±15.36	77.35±19.54^a^	75.48±18.48^a^
HDL-C (mg/dL)	47.83±14.64	61.11±17.20^a^	65.86±15.54^a^
TP (g/dL)	4.56±0.38	4.82±0.49	4.77±0.49
ALB (g/dL)	1.79±0.35	1.85±0.37	1.72±0.31
AST (U/L)	80.70±74.71	76.00±48.06	76.13±42.84
ALT (U/L)	70.71±36.37	52.67±32.62	56.70±28.68
UA (mg/dL)	1.59±4.04	3.07±8.23	6.51±11.77
UN (mg/dL)	26.41±8.82	33.32±27.82	27.56±7.56

All values are presented as mean ± SD. Statistical analysis was performed using Mann-Whitney U test. TG, triglyceride; CHO, cholesterol; HDL-C, high-density lipoprotein; TP, total protein; ALB, albumin; AST, aspartate aminotransferase; ALT, alanine aminotransferase; UA, uric acid; UN, urea nitrogen.

^a^ *p*<0.05 compared to wild-type mice.

### Effect of magnolol on plasma triglyceride level on transgenic knock-in mice

**Fig 4:**
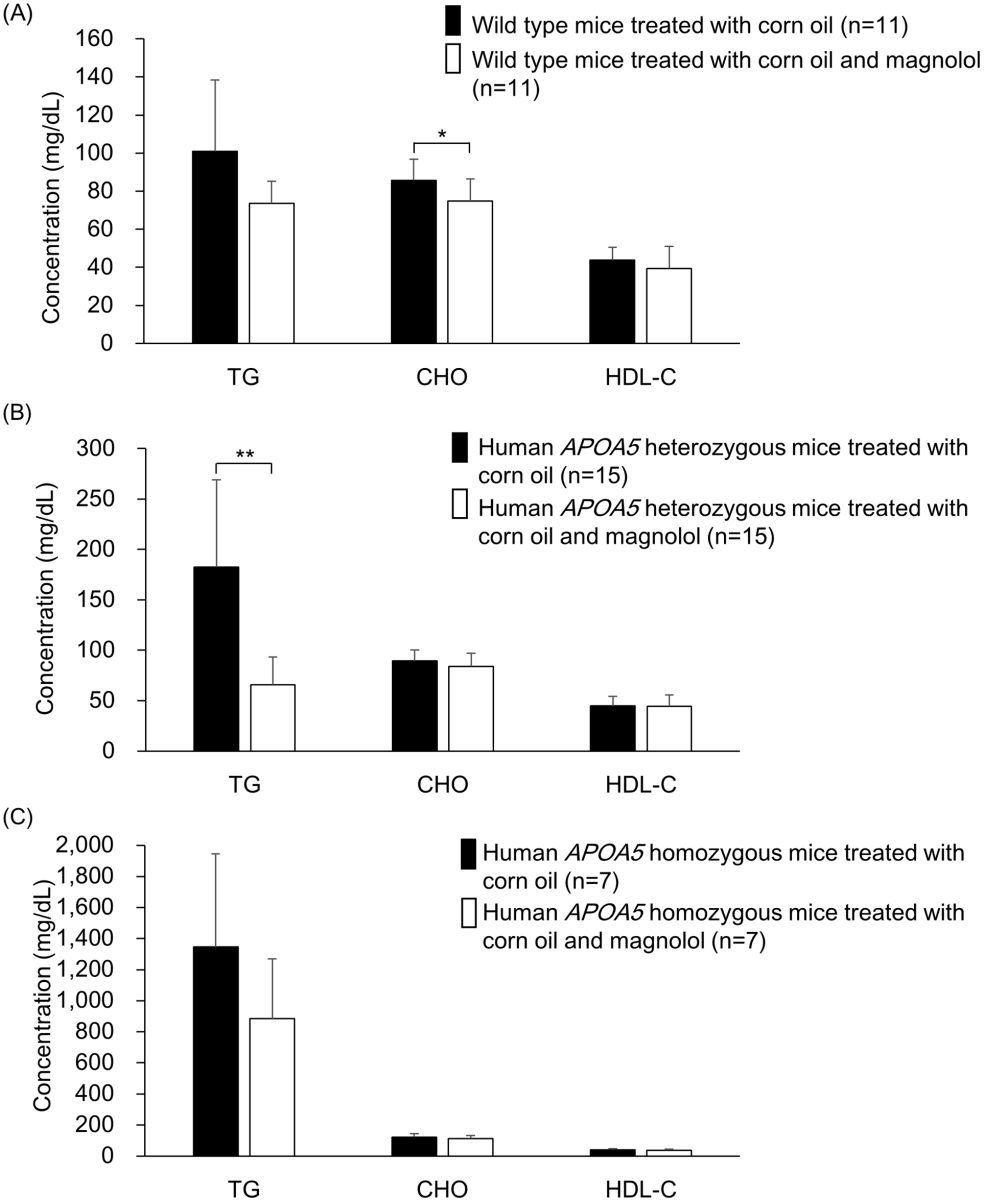
The effect of magnolol on plasma triglyceride level in mice. Human *APOA5* c.553G>T knock-in male 12-week-old B6 mice were divided into wild-type (n = 11) (A), heterozygous (n = 15) (B) and homozygous (n = 7) (C) groups. A corn oil bolus (6 ml/kg) or magnolol (30 mg/kg; 30 μg/μL in corn oil) was administered by oral gavage once daily for 4 days. On the fourth day, 2 h after feeding, knock-in mice were anesthetized using avertin and heparinized blood was collected via inferior vena cava for determination of lipid concentrations. Statistical analysis was performed using Mann-Whitney U test. * *p*<0.05; ** *p*<0.001.

After demonstrating that transgenic knock-in mice carrying human *APOA5* c.553G>T variant had increased plasma triglyceride levels, experiment was conducted to determine the impact of magnolol on lipid metabolism *in vivo*. To investigate whether magnolol could reduce plasma triglyceride levels, 12-week-old transgenic knock-in mice carrying human *APOA5* c.553G>T variant were fed with corn oil (6 mL/kg) with or without magnolol (30 mg/kg) for four days. Mice were anesthetized and blood sample was collected with heparin as anticoagulant. In the absence of magnolol, plasma triglyceride levels in mice carrying one and two alleles of *APOA5* c.553G>T variant increased significantly, up to 180 mg/dL and 1,400 mg/dL, respectively (Fig 4). An apparent reduction of triglyceride level was observed in three groups of mice fed with magnolol, although a significant change was only demonstrated in the heterozygous group (*p*<0.005). In addition, no significant difference of ALT level was observed in all mice fed with magnolol (data not shown).

## Discussion

Our study demonstrates that magnolol could efficiently reduce the postprandial plasma triglyceride level in *APOA5* c.553G>T variant carrier mice and facilitate triglyceride metabolism in mild postprandial hypertriglyceridemia. LPL activity in both cell lysates and supernatant was significantly increased along with the increased magnolol concentrations, and the activity of LPL increased with dose-dependence *in vitro*. We also showed that the mRNA expression level of *LPL* was significantly elevated in the presence of magnolol, and a weak dose-dependence was demonstrated. This indicated that increased LPL activity was probably through enhancing the *LPL* mRNA expression. Surprisingly, the greatest postprandial triglyceride decrease was observed in *APOA5* c.553G>T heterozygous mice. Although the postprandial triglyceride was also decreased in homozygous mice, no statistically significant difference was observed. The reason for this discrepancy may be caused by the incapability of activation of LPL by APOA5 G185C protein. Even induction by magnolol, this incapable protein still could not activate LPL and hydrolyze the high concentrations of triglyceride as reported previously [11]. This is different from that of *APOA5* c.553G>T heterozygous mice which synthesized half of functional APOA5 protein and capably hydrolyzed postprandial hypertriglyceridemia. King et al. observed that the maximal increase in serum triglycerides was observed two hour after corn oil administration and returning towards baseline levels within three hour in normolipidemic CD-1 and C57BL/6 mice [19]. In this study we followed the same protocol and blood was collected two hour after corn oil administration. Magnolol efficiently reduced the serum triglyceride level even at its peak concentration in *APOA5* c.553G>T heterozygous mice. This indicated that the potential application of magnolol in lowering postprandial hypertriglyceridemia. Whether single dose of magnolol administration still has such efficacy in reducing postprandial hypertriglyceridemia is unclear. A further investigation is required.

Fakhrudin et al. have shown magnolol could activate PPARγ and acted as a dual agonist activating PPARβ/δ at higher concentrations [21]. Another report also showed that magnolol enhanced adipocyte differentiation in 3T3-L1 cells, and it was suggested that these effects might be due to PPARγ modulation [14]. Schoonjans et al. reported that transcriptional activation of the LPL gene by activators was mediated by PPAR-RXR heterodimers. All these reports indicated that magnolol could activate PPARγ, leading to the elevated expression of *LPL* gene [22]. We confirmed this mechanism that the enhancement of LPL activity by magnolol was suppressed in the presence of GW9662 alone or GW9662 and MK886 in combination, which inhibited PPARγ and PPARα respectively.

Hypertriglyceridemia may be caused by either overproduction or accumulation of chylomicrons or VLDL in the circulation. Chylomicron increase is generally the result of impaired lipoprotein involvement, while accumulation of VLDL is usually due to the excess lipoprotein input and/or impaired removal. Both chylomicrons and VLDL are changed to remnant lipoproteins through the lipolytic action of LPL and hepatic lipase (HL). Hypertriglyceridemia is a common metabolic disorder, but the causes are not well understood. A number of studies have shown that, in addition to the environmental factors, genetic implication may play an important role in the susceptibility to hypertriglyceridemia. Primary hypertriglyceridemia has been associated with LPL deficiency, apolipoprotein CII deficiency or HL deficiency [23–25]. In addition to the apolipoprotein CIII gene, the influence of polymorphism in the *APOA5* gene on serum triglyceride level has also been reported, although the association is different between ethnicities [7, 8, 26]. In our previous report, we characterized the association between *APOA5* c.553G>T variant and hypertriglyceridemia [9]. We also verified that residue 185G of human APOA5 is indispensable for LPL activation, any variant would reduce LPL activation [11]. This variant is associated with increased risk of cardiovascular disease [27]. Genetic variants causing postprandial hypertriglyceridemia have been reviewed [28–30].

In the current study, we demonstrated that mice carrying human *APOA5* c.553G>T variant had postprandial hypertriglyceridemia. Postprandial hypertriglyceridemia have played a critical role in the pathogenesis of atherosclerosis [31–34]. However, the specific role of triglyceride-rich lipoproteins in atherosclerosis remains uncertainty [35–37]. Epidemiologic studies on non-fasting triglyceride levels also found strong associations between cardiovascular events with increases in non-fasting triglyceride [38–42]. In addition to the increased risk of myocardial infarction and ischemic heart disease, non-fasting TG levels also associated with increased risk of ischemic stroke [43]. Current guidelines do not recommend the target value for the triglyceride of postprandial state, although fibrates, niacin and 3-omega fatty acids have been commonly used to reduce plasma TG, particularly in subjects with high residual risk on statin therapy [35, 37, 44]. However, fibrates are the only available option as additional therapy to statins to reduce the residual risk [45–47], because of the questioned clinical benefits of both niacin and 3-omega fatty acids [48, 49].

Our results are expected to shed light on the potential pharmacological application for magnolol. This human *APOA5* c.553G>T transgenic knock-in mice could be used as platform to study the perturbations in postprandial hypertriglyceridemia due to *APOA5* c.553G>T variant.

## Supporting information

S1 Fig Schematic design of the construction of vector and generation of human *APOA5* knock-in mice. (A) Targeting DNA from BACs was subcloned into a high-copy plasmid vector, pL253 and then electroporated into EL350 *E*. *coli* to generate retrieved A. (B) Construction of retrieved C which contained pL452 with two *lox* P sites on both ends of *Neo* cassette. Insertion of the first *lox*P site into retrieved A to generate retrieved A + vector C—C. (C) Construction of vector B containing pL451 and human *APOA5* mini genes. After digestion, both vector B and retrieved A + vector C—C were electroporated into EL350 *E*. *coli*. After induction of Cre expression by arabinose, the targeting vector A + C—C + B—B was generated. (D) The targeting vector was subsequently linearized and electroporated into CJ7 ES cells. The final selected clones of ES cells were injected into mouse blastocysts (E3.5) to create the chimeric offspring.
(TIF)
