# Supplementary figures and images for: Magnolol-mediated regulation of plasma triglyceride through affecting lipoprotein lipase activity in apolipoprotein A5 knock-in mice
Source: PLoS One. 2018 Feb 9;13(2):e0192740. doi: 10.1371/journal.pone.0192740 (PMC5806881; doi:10.1371/journal.pone.0192740)

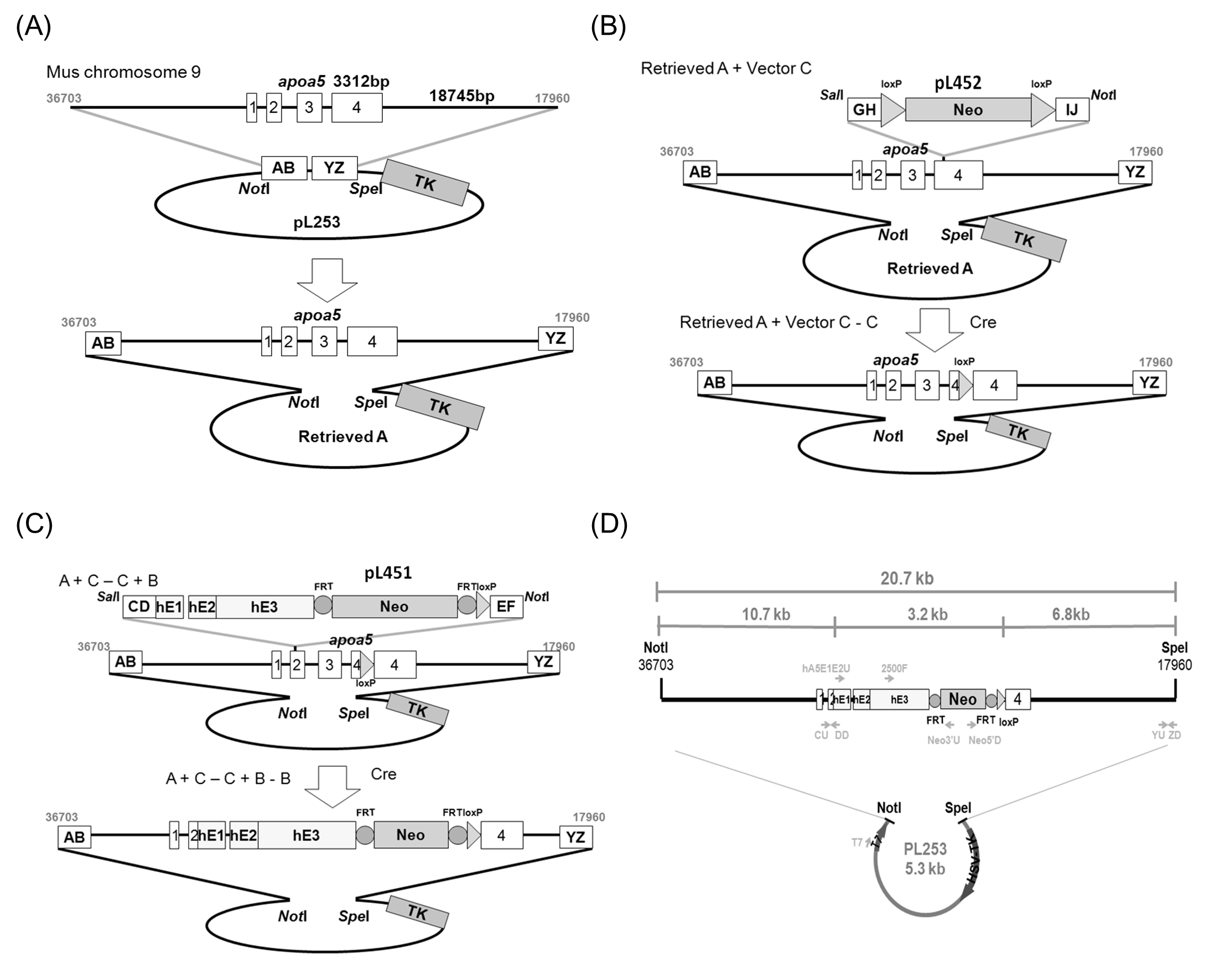

Supplement: S1 Fig — (A) Targeting DNA from BACs was subcloned into a high-copy plasmid vector, pL253 and then electroporated into EL350 E. coli to generate retrieved A. (B) Construction of retrieved C which contained pL452 with two lox P sites on both ends of Neo cassette. Insertion of the first loxP site into retrieved A to generate retrieved A + vector C—C. (C) Construction of vector B containing pL451 and human APOA5 mini genes. After digestion, both vector B and retrieved A + vector C—C were electroporated into EL350 E. coli. After induction of Cre expression by arabinose, the targeting vector A + C—C + B—B was generated. (D) The targeting vector was subsequently linearized and electroporated into CJ7 ES cells. The final selected clones of ES cells were injected into mouse blastocysts (E3.5) to create the chimeric offspring. (TIF) [file pone.0192740.s001.tif]
